# Common *versus* psychopathology-specific risk factors for psychotic experiences and depression during adolescence

**DOI:** 10.1017/S0033291714000026

**Published:** 2014-01-31

**Authors:** D. Kounali, S. Zammit, N. Wiles, S. Sullivan, M. Cannon, J. Stochl, P. Jones, L. Mahedy, S. H. Gage, J. Heron, G. Lewis

**Affiliations:** 1 School of Social and Community Medicine, University of Bristol, UK; 2 MRC Centre for Neuropsychiatric Genetics and Genomics, Cardiff University, Wales, UK; 3 Royal College of Surgeons in Ireland and Beaumont Hospital, Dublin, Ireland; 4 Department of Psychiatry, University of Cambridge, UK

**Keywords:** ALSPAC, depression, epidemiology, psychotic experiences, risk factors

## Abstract

**Background:**

An argument often used to support the view that psychotic experiences (PEs) in general population samples are a valid phenotype for studying the aetiology of schizophrenia is that risk factors for schizophrenia show similar patterns of association with PEs. However, PEs often co-occur with depression, and no study has explicitly tested whether risk factors for schizophrenia are shared between PEs and depression, or are psychopathology specific, while jointly modelling both outcomes.

**Method:**

We used data from 7030 subjects from a birth cohort study. Depression and PEs at age 18 years were assessed using self-report questionnaires and semi-structured interviews. We compared the extent to which risk factors for schizophrenia across sociodemographic, familial, neurodevelopmental, stress–adversity, emotional–behavioural and substance use domains showed different associations with PEs and depression within bivariate models that allowed for their correlation.

**Results:**

Most of the exposures examined were associated, to a similar degree, with an increased risk of both outcomes. However, whereas female sex and family history of depression showed some discrimination as potential risk factors for depression and PEs, with stronger associations in the former, markers of abnormal neurodevelopment showed stronger associations with PEs.

**Conclusions:**

The argument that PEs are valid markers for studying the aetiology of schizophrenia, made simply on the basis that they share risk factors in common, is not well supported. PEs seem to be a weak index of genetic and environmental risk for schizophrenia; however, studies disentangling aetiological pathways to PEs from those impacting upon co-morbid psychopathology might provide important insights into the aetiology of psychotic disorders.

## Introduction

Psychotic experiences (PEs) such as delusions and hallucinations can be elicited using semi-structured interviews in approximately 5% of adolescents and young adults in the general population (Linscott & van Os, 2013). Delusions and hallucinations are key diagnostic features of schizophrenia, and studying PEs may increase our understanding of how schizophrenia develops if PEs in general population samples result from aetiological mechanisms underlying schizophrenia.

PEs during childhood are associated with an increased risk of schizophrenia spectrum and other psychotic disorders during adult life (Hanssen *et al.* 2005; Welham *et al.* 2009*b*; Werbeloff *et al.* 2012; Fisher *et al.* 2013; Zammit *et al.* 2013*b*), but the positive predictive value of such experiences is low (Poulton *et al.* 2000; Dhossche *et al.* 2002; Dominguez *et al.* 2011; Werbeloff *et al.* 2012; Zammit *et al.* 2013*b*). Consequently, evidence often used to support the view that PEs constitute a valid phenotype for studying the aetiology of schizophrenia is that risk factors for schizophrenia (Malmberg *et al.* 1998; Mortensen *et al.* 1999; Cannon *et al.* 2002*b*; McGrath *et al.* 2004; Wicks *et al.* 2005; Moore *et al.* 2007; Morgan & Fisher, 2007; van Os & Kapur, 2009; Welham *et al.* 2009*a*) show similar patterns of association with PEs, with reports that PEs are associated with male sex (van Os *et al.* 2009; Kelleher *et al.* 2012*a*), family history of schizophrenia (Krabbendam *et al.* 2004; Polanczyk *et al.* 2010), migrant or ethnic minority status (van Os *et al.* 2009), urbanicity (Dominguez *et al.* 2010; Polanczyk *et al.* 2010), socio-economic disadvantage (van Os *et al.* 2009; Polanczyk *et al.* 2010; Zammit *et al.* 2013*b*), pregnancy and birth complications (Zammit *et al.* 2009), developmental impairments (Cannon *et al.* 2002*a*), lower IQ (Cannon *et al.* 2002*a*; Horwood *et al.* 2008), stressful and traumatic experiences (Arseneault *et al.* 2011), peer victimization (Schreier *et al.* 2009), poorer social adjustment (Polanczyk *et al.* 2010) and cannabis use (Moore *et al.* 2007).

However, the overlap in the epidemiology of PEs with that for schizophrenia is not entirely consistent, with some studies reporting that PEs are observed more frequently in women (van Os *et al.* 2000; Breetvelt *et al.* 2010; Binbay *et al.* 2012; Nuevo *et al.* 2012; Zammit *et al.* 2013*b*) or in those living in rural areas (Wiles *et al.* 2006; Bartels-Velthuis *et al.* 2010), or failing to find evidence of increased risk of PEs in those with a family history of schizophrenia (Zammit *et al.* 2008). Even where epidemiological findings for schizophrenia and PEs are consistent, it is questionable to what extent these demonstrate evidence of shared aetiology, given that most of the factors associated with increased risk of schizophrenia and PEs are also associated with other psychiatric outcomes. For example, socio-economic adversity (Lorant *et al.* 2003), pregnancy and birth complications (Nosarti *et al.* 2012), developmental delay and cognitive impairment (van Os *et al.* 1997), autistic traits (Lundstrom *et al.* 2011), emotional problems and interpersonal difficulties during childhood (Cannon *et al.* 2002*a*), stressful or traumatic events and victimization (Thapar *et al.* 2012) and cannabis use (Moore *et al.* 2007) are also associated with increased risk of depression.

In fact, co-morbidity with depression is one of the most consistent findings regarding PEs in general population samples (Dhossche *et al.* 2002; Scott *et al.* 2009; Armando *et al.* 2010; van Rossum *et al.* 2011; Varghese *et al.* 2011; Kelleher *et al.* 2012*a*). One interpretation of this is that PEs might be a manifestation of depression rather than an indication of abnormal pathology underlying schizophrenia-related disorders. Understanding how best to conceptualize PEs is important because this has implications for classification of PEs and for predicting their utility in aiding our understanding of mechanisms underlying the aetiology of schizophrenia.

Studies examining latent constructs of psychopathology show that depression and psychosis exist as separate, but strongly correlated, dimensions in the general population (Stefanis *et al.* 2002; Krabbendam *et al.* 2004). Given the degree of co-morbidity, it is therefore not unexpected that most risk factors for PEs are also associated with risk of depression, as described earlier. Although some studies have examined risk factors for both depression and PEs within the same study (Krabbendam *et al.* 2004; Breetvelt *et al.* 2010), as far as we are aware no study has explicitly tested whether risk factors are shared between PEs and depression or are psychopathology specific while jointly modelling both outcomes to account for their co-morbidity. Examining to what extent risk factors for schizophrenia show similar, or different, patterns of association with PEs compared to depression can help to shed light on how useful studies of PEs might be in helping us understand the aetiology of schizophrenia.

In this study we compared the extent to which risk factors for schizophrenia across sociodemographic, familial, neurodevelopmental, stress and adversity, emotional–behavioural and substance use domains show different associations with PEs compared to with depression. We used statistical methods that account for the correlation between PEs and depression to avoid the problem of similar associations for PEs and depression resulting from co-morbidity of these two outcomes.

## Method

### Participants

The sample comprised participants from the Avon Longitudinal Study of Parents and Children (ALSPAC; www.alspac.bris.ac.uk), an ongoing population-based study investigating a wide range of influences on health and development of children (Boyd *et al.* 2012). Ethical approval for the study was obtained from the ALSPAC Law and Ethics Committee and the Local Research Ethics Committees. From the initial core sample of 14 541 pregnancies, 13 988 children were alive at 12 months. Among these, 7030 children had at least one measure of interest (depression or PE measures at either childhood or early adulthood) and were included in the analysis.

### Measures

#### Depression outcome at 18 years

The depression outcome measure at 18 years used in this study was based on the computerized revised Clinical Interview Schedule (CIS-R; Lewis *et al.* 1992; Lewis, 1994), a fully structured interview designed for community samples. The computerized version shows very close agreement with the interviewer-administered version (Patton *et al.* 1999; Bell *et al.* 2005). The assessment asks questions about the week prior to interview and the onset and duration of each episode. The depression symptom score reflects the severity of depression symptoms and was calculated as the sum of the scores for the following CIS-R items: depression, depressive thoughts, fatigue, concentration and sleep (score range: 0–21). As depression can be considered as a continuum, we used a cut-off of 10 to indicate the presence of depression. Selection of this cut-off resulted in a prevalence of cases that was the same as the prevalence of PEs, thus aiding comparison of probit scores and odds ratios (ORs) for these two outcomes.

#### PEs at 18 years

PEs at 18 years were assessed using the Psychosis-Like Symptom interview (PLIKSi; Horwood *et al.* 2008; Zammit *et al.* 2013*b*). The PLIKSi is a semi-structured instrument that draws on the principles of standardized clinical examination developed for the Schedule for Clinical Assessment in Psychiatry (SCAN) and covers hallucinations (visual and auditory), delusions (spied on, persecution, thoughts read, reference, control, grandiosity) and experiences of thought interference (broadcasting, insertion and withdrawal). Any unspecified delusions elicited were also rated. Cross-questioning was used to establish the presence of symptoms, and coding followed glossary definitions and rating rules for the SCAN. Interviewers rated experiences as not present, suspected or definitely psychotic that had occurred since age 12 years. Unclear responses after probing were always ‘rated down’, and symptoms only rated as definite when a clear example was provided. PEs were coded as present in this study if one or more of the experiences was either ‘suspected or definitely present’ and if they could not be directly attributed to falling asleep/waking or to fever.

#### Exposures

Based on evidence from previous literature that these were associated with schizophrenia, we examined 19 risk factors across the following six domains (see Table 1 and Supplementary online Appendix 1 for more detail):

(1) Sociodemographic: gender, ethnicity, maternal education, maternal marital status, maternal home ownership, urbanicity.
(2) Familial: family history of depression and family history of schizophrenia.
(3) Neurodevelopmental: hypoxia at birth, gross motor development (and change) assessed at 6 and 18 months, cognitive function at 8 years, and autistic spectrum traits characterized between 6 months and 9 years.
(4) Stress–adversity: life events at 9 years and victimization at 8 years.
(5) Emotional–behavioural: Strength and Difficulties Questionnaire (SDQ) domains for conduct, hyperactivity–inattention and peer relationship problems; the Development and Well Being Assessment (DAWBA) for anxiety at age 10; the Short Mood and Feelings Questionnaire (SMFQ) for depressive symptoms at 12 years; the PLIKSi for PEs at 12 years.
(6) Substance use: cannabis use up to the age of 16 years.

**Table 1.** Description of exposures under study

Measure	Scale	Analysis units	Sample *n*	*n* (%) exposed
Sociodemographic				
Ethinicity	Binary	Non-white	6641	277 (4.2)
Urbanicity	Binary	Rural	6958	449 (6.5)
Sex	Binary	Female	7030	3626 (51.6)
Marital status of mother	Binary	Not married	6866	1292 (18.8)
Low maternal education	Binary	CSEs	6504	662 (9.42)
Home ownership	Binary	Renting	6800	1164 (17.12)
Familial				
Family history of depression	Binary	Present	6749	1922 (28.5)
Family history of schizophrenia	Binary	Present	5773	166 (2.88)
Neurodevelopmental				
Hypoxia	Binary	Present	6449	558 (8.7)
Gross motor score at 6 months	*z* score^a^	s.d. scale	6067	
Gross motor score at 18 months	*z* score^a^	s.d. scale	6297	
IQ at 8 years	WISC (45–151)	NED (1 s.d. = 15)^b^	5811	
Autistic spectrum disorder	Binary	< –1	6957	1104 (15.7)
Stress and adversity				
Life events at 7 years	0, 1, ⩾ 2	0	5882	4210 (71.6)
		1		1376 (23.4)
		⩾ 2 events		296 (5.0)
Victimization at 8 years	Binary	Present	5517	2164 (39.2)
Emotional–behavioural				
Conduct score at 12 years	0–10	1 s.d. = 2^a^	5662	
Hyperactivity score at 12 years	0–10	1 s.d. = 2^a^	5651	
Peer problems score at 12 years	0–10	1 s.d. = 2^a^	5663	
Depression at 12 years	0–26	> 11 score	6344	579 (9.13)
Anxiety disorder at 10 years	Binary	Present	5774	110 (1.91)
PEs at 12 years	Binary	Present	6448	745 (11.6)
Substance use				
Cannabis use at 16 years	Lifetime count	Never	4102	2920 (71.2)
		< 20 times		854 (20.8)
		20–60		159 (3.9)
		> 60		169 (4.12)

CSE, Certificate of Secondary Education; WISC, Wechsler Intelligence Scale for Children; NED, normal equivalent deviates; PE, psychotic experience.

a Analysis scale is expressed in standard deviation (s.d.) units where the scale is transformed to have a mean 0 and s.d. = 1.

b NED – standardized scale. Raw scores are transformed to *z* scores (standard normal scores) using the inverse normal function.

### Statistical analysis

Bivariate probit regression models were used to jointly model the binary indicators of depression and PE at 18 years and to test for equality of regression parameters expressing the effect of exposures on each outcome using likelihood ratio tests. For this purpose we compared a model that allowed effect estimates to differ for our outcomes with a model where the exposure effect was constrained to be the same for both outcomes. We converted probit estimates into ORs to enable presentation of results in a format that would be more easily interpretable. This was achieved by obtaining approximations of the logit parameters by multiplying the probit parameters by a factor of 1.6 (see Amemiya 1981, eq. 2.7; Ntzoufras *et al.* 2003). As a sensitivity analysis, we also examined the effect of exposures (omitting cannabis use and previous measures of PEs and depression) on a multivariate outcome that used data on these outcomes across both ages 12 and 18.

There were significant amounts of missing data on both outcomes. Missing depression or PE outcome data at 18 years was strongly associated with gender, socio-economic position and early loss of follow-up. To deal with missing data, we used multiple imputation (MI; Little & Rubin, 2002) by fully conditional specification using flexible additive imputation models as implemented in the aregImpute function in the R statistical package (Harell, 2001; van Buuren *et al.* 2006). In addition to those included in the analyses, the imputation model also included variables that were associated with missingness or were predictive of depression or PE at 18 years. Approximately 60 auxiliary variables were included in the imputation model to make the basic assumption underlying MI realistic. These included a wide range of markers of sociodemographic characteristics during early childhood and historical assessments of most exposures examined (e.g. SDQ scores were assessed repeatedly at ages 47 and 81 months, and 8, 9, 10, 11 and 13 years). Further information is available from the authors. Parameter estimates were averaged over 100 imputed datasets using Rubin rules. Wald-type tests were used following imputation to assess equality or commonality of exposure effects.

## Results

### Descriptive

Of the 7030 individuals in the analysis sample, 4140 had data on PEs at age 18 years. Of these, 370 [9.0%, 95% confidence interval (CI) 8.2–9.9] had at least one PE present (see Zammit *et al.* 2013*b* for further details). Of 4435 individuals who had data on depression at age 18 years, 410 (9.2%, 95% CI 8.4–10.1) had a depression score of > 10 (cut-off selected to ensure similar prevalence of both outcomes). Among those with PEs at age 18 years, 28% also had depression; and among those with depression, 28% also had PEs.

The correlation between depression and PE was 0.20 (95% CI 0.09–0.31) at age 12 and 0.22 (95% CI 0.09–0.36) at age 18, and this co-occurrence was sustained over time from late childhood to early adulthood (*r* = 0.72, 95% CI 0.61–0.82).

### Common and specific risk factors

The results for the association between each exposure and both depression and PEs at age 18 are summarized in Table 2. Most of the exposures examined within the sociodemographic, stress and adversity, emotional–behavioural and substance use risk domains were associated, to a similar degree, with an increased risk of both outcomes. This was true for low maternal education, socio-economic disadvantage and single parenthood at birth, stressful life events, peer victimization, anxiety disorder, hyperactivity, conduct problems, peer problems during childhood, and cannabis use during adolescence.

**Table 2.** Exposure effects (OR and 95% CI) on depression and psychotic experiences (PEs) at 18 years^a^, and examination of whether psychopathology-specific effects differ from a common effect

	*n*	Depression		PEs		Common effect		
		OR	95% CI	OR	95% CI	OR	95% CI	*p* value^b^
Sociodemographic								
Ethnicity (non-White)	3916	1.42	0.95–2.12	1.44	0.97–2.16	1.43	1.05–1.96	0.942
Urbanicity (rural)	4060	0.90	0.64–1.28	0.73	0.50–1.05	0.81	0.62–1.07	0.347
Sex (female)	4100	1.99	1.64–2.38	1.34	1.12–1.60	1.61	1.41–1.86	< 0.001
Maternal status (unmarried)	4035	1.58	1.28–1.95	1.74	1.41–2.14	1.66	1.41–1.95	0.467
Maternal education (low)	3847	1.27	0.94–1.71	1.57	1.18–2.09	1.42	1.13–1.78	0.253
Home ownership (rented)	3988	1.60	1.28–1.99	1.67	1.34–2.08	1.63	1.37–1.94	0.745
Familial								
Family history of depression	3970	1.45	1.21–1.75	1.10	0.91–1.34	1.27	1.10–1.47	0.023
Family history of schizophrenia	3571	1.57	0.98–2.53	1.33	0.81–2.18	1.45	0.99–2.12	0.584
Neurodevelopmental								
Asphyxia	3766	0.79	0.56–1.11	1.30	0.96–1.75	1.03	0.81–1.32	0.015
Gross motor levels	3402	1.04	0.98–1.11	1.08	1.02–1.15	1.06	1.01–1.11	0.323
Gross motor differentials	3402	0.98	0.90–1.06	1.12	1.03–1.22	1.05	0.98–1.11	0.011
IQ	3498	1.00	0.91–1.10	0.88	0.79–0.97	0.94	0.87–1.01	0.037
Autistic spectrum disorder	4100	0.89	0.70–1.14	1.29	1.03–1.61	1.08	0.90–1.03	0.016
Stress and adversity								
Life events	3525	1.14	0.99–1.32	1.13	0.98–1.32	1.14	1.01–1.27	0.952
Victimization	3339	1.42	1.17–1.72	1.50	1.23–1.83	1.46	1.25–1.70	0.659
Emotional–behavioural								
Conduct	3389	1.24	1.13–1.37	1.27	1.15–1.41	1.26	1.16–1.36	0.704
Hyperactivity	3388	1.11	1.01–1.23	1.21	1.09–1.03	1.16	1.07–1.25	0.209
Peer problems	3390	1.19	1.08–1.31	1.20	1.09–1.33	1.20	1.11–1.29	0.874
Anxiety	3452	1.36	0.71–2.62	1.82	0.98–3.36	1.58	0.96–2.60	0.474
Depression (at 12 years)	3538	3.41	2.63–4.42	2.71	2.07–3.54	3.05	2.48–3.75	0.173
PEs (at 12 years)	3585	2.36	1.84–3.02	4.06	3.20–5.15	3.12	2.59–3.76	< 0.001
Substance use								
Cannabis use	2959	1.23	1.11–1.36	1.21	1.09–1.34	1.22	1.13–1.32	0.765

OR, Odds ratio; CI, confidence interval.

a Complete case data, bivariate model.

b *p* values associated with significance tests comparing a model assuming psychopathology-specific effect for each exposure *versus* a model where the exposure effect is common/shared (i.e. constrained to be the same across psychopathologies). Small *p* values indicate evidence of differences in fit between the two models, whereby the shared-effect model does not provide adequate fit for the data and a psychopathology-specific model provides a better fit.

Most measures of neurodevelopment, including asphyxia at birth, early motor developmental delay, lower IQ and autistic traits, were associated with an increased risk of PEs but not depression. The likelihood ratio tests for these exposures provide support for the notion that modelling exposure effects as psychopathology specific provides a better fit to the data than modelling a common effect across depression and PEs. There was also strong evidence that PEs at age 12 were more strongly associated with PEs at age 18 than with depression at this age.

By contrast, depression at age 12 led to an approximately threefold increase in risk of both depression and PEs at age 18, whereas family history of depression and female sex in particular were more strongly associated with depression than PEs at age 18.

### MI

Data for depression and PEs were both missing at age 12 years in 8% (*n* = 555) of the 7030 individuals in the analysis sample, and in 37% (*n* = 2595) at age 18 years. Approximately 50% (*n* = 3524) had data on both depression and PE outcomes at age 12 and age 18 years. Approximately 36% (*n* = 2519) of those with data on both depression and PE at age 12 did not have any outcome data at 18 years, whereas only 7% (*n* = 501) of those with both depression and PE data at age 18 did not have any data on either measure at age 12. Thus the main pattern for missingness was loss of follow-up. Pooled estimates from MI were similar to those using the complete-case data, and conclusions were essentially unchanged (see online Supplementary Table S1). The results were also substantively the same when examining a multivariate outcome using data on PEs and depression across both ages 12 and 18 (Supplementary Table S2).

## Discussion

In this study we found evidence that most of the established risk factors for schizophrenia that we examined were associated with both PEs and depression during late adolescence within a general population sample, even after allowing for the co-morbidity between these two outcomes. Most of the exposures examined within the sociodemographic, stress and adversity, emotional–behavioural and substance use risk domains were associated, to a similar degree, with an increased risk of both outcomes. However, whereas female sex and, to a lesser extent, family history of depression showed some discrimination as potential risk factors for depression and PEs, occurring more commonly in the former, markers of abnormal neurodevelopment discriminated between these phenotypes, with stronger associations observed for PEs.

### Previous studies

Studies that have examined risk factors for both PEs and depression have not been able to make direct comparisons of effects across these outcomes. For example, one cross-sectional study examined associations between sociodemographic characteristics and outcomes of psychotic symptoms and of depression while adjusting for (or conditioning on) the other outcome (Breetvelt *et al.* 2010). Although there was statistical evidence that ethnicity and markers of sociodemographic adversity were associated with both outcomes, and that female sex was associated with depression but not PEs, the models used do not permit formal testing of whether risk factors are shared, and to a similar extent, across both outcomes or are disorder specific. Similarly, another study (Krabbendam *et al.* 2004) that examined associations between risk factors and latent constructs of depression and psychosis in the general population did not test whether associations were different across these outcomes, or model these outcomes jointly to allow for the correlation between them. Given the high levels of co-morbidity between depression and PEs in general population samples (Kelleher *et al.* 2012*a*), statistical approaches that can test for differences between risk factor associations on these outcomes while allowing for their strong correlation are important.

Consistent with our findings, there is strong evidence that risk of schizophrenia is increased in individuals with neurodevelopmental abnormalities. Longitudinal studies have demonstrated that lower IQ during childhood and adolescence is associated with an increased incidence of psychotic disorders (Khandaker *et al.* 2011; Dickson *et al.* 2012), with similar findings observed for markers of motor and language development during early childhood (Welham *et al.* 2009*a*; Dickson *et al.* 2012). A wide variety of pregnancy and birth complications, including hypoxia-related exposures, have been associated with risk for schizophrenia (Cannon *et al.* 2002*b*; Welham *et al.* 2009*a*), whereas genetic studies have demonstrated substantial overlap between the genetic aetiology for schizophrenia with that for autism and other neurodevelopmental disorders (Williams *et al.* 2010; Doherty *et al.* 2012). Although there is also some evidence that markers of impaired neurodevelopment (van Os *et al.* 1997; Zammit *et al.* 2004; Gale *et al.* 2008, 2009), autistic traits (Lundstrom *et al.* 2011) and birth complications (Nosarti *et al.* 2012) are associated with risk of depression, these results have been weaker and less consistent than those for schizophrenia (see, for example, Cannon *et al.* 2002*a*), but again no studies have directly compared associations with schizophrenia and depression within bivariate models that allow for co-morbidity of these disorders.

### Interpretation of findings

Our finding, that markers of neurodevelopmental impairment are more strongly associated with PEs than with depression within such a model, supports the belief that these experiences are aetiologically related to pathological mechanisms underlying schizophrenia, and that their study might help us to understand more about neurodevelopmental mechanisms in the aetiology of this disorder. However, most of the exposures examined were associated with depression and PEs to the same extent. The argument that PEs are valid markers for studying the aetiology of schizophrenia, made simply on the basis that they share risk factors in common with each other, is therefore not well supported. Furthermore, although neurodevelopmental abnormalities and sex show some discrimination between PEs and depression, it is feasible that these characteristics do not discriminate PEs from other psychopathologies, such as attention deficit hyperactivity disorder, that were not examined here. Evidence supports the presence of an extended phenotype for psychosis existing across several dimensions, including an affective domain (David, 2010; Kaymaz & van Os, 2010). Further examination of the extent to which specific characteristics distinguish PEs from other psychopathologies could lead to a greater understanding of the aetiology of psychosis.

### Strengths and limitations

One of the strengths of our study is that we were able to examine and compare the effects of a wide range of risk factors measured longitudinally with respect to jointly modelled outcomes of depression and PEs. Although other studies of PEs in general population samples have described associations with IQ (Cannon *et al.* 2002*a*; Horwood *et al.* 2008), developmental delay (Cannon *et al.* 2002*a*), autistic traits (Bevan Jones *et al.* 2012) and birth complications (Zammit *et al.* 2009), none of these have examined whether associations with PEs are different from those for depression within multivariate models that take into account the co-morbidity or correlation of these outcomes.

One of the main limitations of our study was the substantial attrition in the cohort over time. However, the results were very similar in the imputation model that included measures of the family's socio-economic status, lifestyle and mental health, and several measures of the child's psychosocial development including data from earlier waves of depressive symptoms, PEs and other emotional and behavioural problems. Thus the assumption of missing at random conditional on these is not unrealistic.

Another limitation is that we were not able to examine all risk factors for schizophrenia, thus restricting the inferences we can make from this study. Some of the exposures examined such as anxiety disorders and family history of schizophrenia were uncommon, and the power to detect differences across psychopathologies may have been limited. Furthermore, we are not able to determine whether the associations reported here are causal as we have not explored the extent to which these associations are confounded by other variables. However, the aim of this study was not to examine causal relationships, but to explore whether characteristics relating to sociodemographic, developmental and childhood background differed between individuals with PEs and those with depression while allowing for their co-morbidity.

### Implications

Longitudinal studies have demonstrated that the positive predictive value of PEs predicting even a broad phenotype of psychotic disorders is low (Poulton *et al.* 2000; Dhossche *et al.* 2002; Dominguez *et al.* 2011; Werbeloff *et al.* 2012; Zammit *et al.* 2013*b*), and this is likely to be even lower for a rarer outcome such as schizophrenia, whereas there is no evidence that PEs in population-based samples of adolescents share a genetic architecture comparable to schizophrenia (Zammit *et al.* 2013*a*). In keeping with these findings, most of the characteristics we examined, chosen for their consistent associations with schizophrenia in the literature, were associated equally strongly with depression as they were with PEs. Although PEs in the general population may have some clinical utility, given their association with depression (van Rossum *et al.* 2011), suicidality (Kelleher *et al.* 2012*b*) and impaired functioning (Rossler *et al.* 2007), in the main they seem to be a weak index of genetic and environmental risk for schizophrenia.

However, our finding that markers of neurodevelopmental impairment are more strongly associated with PEs than with depression lends some support to the belief that these phenotypes are not just alternative expressions of a common underlying mechanism, and that PEs may share some aetiological mechanisms with schizophrenia. Given their frequency and relative ease of tracking in birth cohort studies, PEs might therefore be a useful phenotype to examine mechanisms by which exposures during early brain development could impact upon risk for schizophrenia. Identification of markers that differentiate PEs from other psychopathology may enable clinicians and researchers to identify individuals with substantially higher risk of transition from PEs to clinical psychotic disorder, and where closer monitoring and earlier intervention might be warranted. More detailed measures of psychopathology over time and analytical methods appropriate for maximizing use of such data are needed to disentangle aetiological pathways to psychosis from those impacting upon co-morbid psychopathology.

## Supplementary Material

Supplementary Material Supplementary information supplied by authors.

Supplementary Material Supplementary information supplied by authors.

## Supplementary material

For supplementary material accompanying this paper visit http://dx.doi.org/10.1017/S0033291714000026.
