# Supplementary material for: Common versus psychopathology-specific risk factors for psychotic experiences and depression during adolescence
Source: Psychol Med. 2014 Jan 31;44(12):2557–66. doi: 10.1017/S0033291714000026 (PMC4108252; doi:10.1017/S0033291714000026)
Supplement: Supplementary Material — Supplementary information supplied by authors. [file S0033291714000026sup001.doc]

**SUPPLEMENTARY MATERIAL**

**Appendix 1: Description of exposures**

**Socio-demographic**

Sex: Male (baseline) *versus* female

Ethnicity: White (baseline) *versus* non-white

Maternal education: Mother’s highest qualification; Vocational/O-levels/A-levels/Degree (baseline) *versus* CSEs (lowest qualifications)

Maternal marital status: Measured during pregnancy with study child; married (baseline) *versus* single/unmarried

Maternal home ownership: Measured during pregnancy with study child; Own home/mortgaged (baseline) *versus* renting

Urbanicity: At birth of study child; city/town (baseline) *versus* village/hamlet

**Familial**

Family history of depression: Depression in parents or grandparents of study child

Family history of schizophrenia: Schizophrenia in parents, aunts, uncles, siblings, or grandparents of study child

**Neurodevelopmental**

Hypoxia at birth: From computerized maternity records; No hypoxia (baseline) *versus* hypoxia (defined as receiving either positive pressure respiratory support (using a face mask or endotracheal tube) or cardiac compressions)

Gross motor development (and change) assessed at 6, 18 months: Gross motor development adapted from the Denver Developmental Screening test covering skills such as kicking and throwing a ball, jumping, and climbing stairs (Dewey *et al.*, 1998, Frankenburg *et al.*, 1996).

Cognitive function at 8 years: The total IQ score was derived from using the WISC-III (Wechsler *et al.*, 1992).

Autistic spectrum traits characterised between 6 months and 9 years: Autistic Spectrum Disorder (ASD) mean factor score was developed within the ALSPAC cohort study in order to identify and characterize correlates of the broader autism phenotype. The ASD mean factor score took into account 93 individual measures reflecting aspects of social, communication and repetitive behaviours characterizing ASD between the ages of 6 months and 9 years. We considered score values (<-1) (Figure 2 in (Steer *et al.*, 2010)) found to characterize the range in the spectrum associated with childhood autism, atypical autism and Asperger syndrome.

**Stress and adversity**

Life events at 9 years: Event counts included whether the child was taken into care; was physically hurt; or sexually abused; was separated from mum and dad; changed caretaker and whether someone close in the family died.

Victimization at 8 years: This was assessed using the Bullying and Friendship Interview Schedule (Schreier *et al.*, 2009); coded as absent (baseline) versus present (presence of any victimization (overt and/or relational))

**Emotional and behavioural**

Strength and Difficulties Questionnaire (SDQ) domains for conduct, hyperactivity-inattention and peer relationship problems: The SDQ (Goodman, 2001) is a behavioural screening questionnaire for children 4-16 years. The 25 items describing positive and negative attributes of children can be allocated to five subscales with five items each: emotional symptoms, conduct problems, hyperactivity-inattention problems, peer relationship problems and prosocial behaviour. The latter was not examined as there is no evidence that this associated with schizophrenia, whilst we used the MFQ (see below) as a measure of emotional symptoms.

The Development and Well Being Assessment (DAWBA) for anxiety at age 10: This is a structured interview aimed at eliciting psychiatric diagnostic information according to ICD-10 among children in the age range of 5 to 16 years (Goodman *et al.*, 2000).

The Short Mood and Feelings Questionnaire (SMFQ) for depressive symptoms at 12 years: The SMFQ is a well-validated tool for assessment of the occurrence of depressive symptoms over the past two weeks (Angold *et al.*, 1995).

**Substance use**

Cannabis use up to the age of 16 years: This was assessed by questionnaire. Individuals were asked about their total cumulative use of cannabis up to the time of the assessment.

**References:**

**Angold A, Costello EJ, Messer SC, Pickles A, Winder F & Silver D** (1995). The development of a short questionnaire for use in epidemiological studies of depression in children and adolescents. *Journal of Methods in Psychiatric Research* **5**, 237-249.

**Dewey C, Fleming P & Golding J** (1998). Does the supine sleeping position have any adverse effects on the child? II. Development in the first 18 months. ALSPAC Study Team. *Pediatrics* **101**, E5.

**Frankenburg WK, Dodds J, Archer P, Bresnick B, Maschka P, Edelman N & Shapiro H** (1996). *Denver II technical manual*. Denver Developmental Materials: Denver, CO.

**Goodman R** (2001). Psychometric properties of the strengths and difficulties questionnaire. *J Am Acad Child Adolesc Psychiatry* **40**, 1337-45.

**Goodman R, Ford T, Richards H, Gatward R & Meltzer H** (2000). The Development and Well-Being Assessment: description and initial validation of an integrated assessment of child and adolescent psychopathology. *J Child Psychol Psychiatry* **41**, 645-55.

**Schreier A, Wolke D, Thomas K, Horwood J, Hollis C, Gunnell D, Lewis G, Thompson A, Zammit S, Duffy L, Salvi G & Harrison G** (2009). Prospective study of peer victimization in childhood and psychotic symptoms in a nonclinical population at age 12 years. *Arch Gen Psychiatry* **66**, 527-36.

**Steer CD, Golding J & Bolton PF** (2010). Traits contributing to the autistic spectrum. *PLoS One* **5**, e12633.

**Wechsler D, Golombock S & Rust J** (1992). *WISC-III UK Weschler Intelligence Scale for Children*. The Psychological Corporation: Sidcup.
