# Supplementary material for: Common versus psychopathology-specific risk factors for psychotic experiences and depression during adolescence
Source: Psychol Med. 2014 Jan 31;44(12):2557–66. doi: 10.1017/S0033291714000026 (PMC4108252; doi:10.1017/S0033291714000026)
Supplement: Supplementary Material — Supplementary information supplied by authors. [file S0033291714000026sup002.doc]

**Supplementary Table S1:** Pooled estimates from multiple imputation (N = 7030) of exposure effects (OR and 95% CI) on depression and psychotic experiences (PEs) at 18 years, and examination of whether psychopathology-specific effects differ from a common effect across psychopathologies

|  | **Depression** | |  | **PEs** | |  | **Common effect** | | |
| --- | --- | --- | --- | --- | --- | --- | --- | --- | --- |
|  | **OR** | **95% CI** |  | **OR** | **95% CI** |  | **OR** | **95% CI** | **P-value^a^** |
| **Sociodemographic** |  |  |  |  |  |  |  |  |  |
| Ethnicity (non-White) | 1.43 | (0.98, 2.08) |  | 1.33 | (0.92, 1.94) |  | 1.38 | (1.04, 1.84) | 0.781 |
| Urbanicty (rural) | 0.86 | (0.61, 1.21) |  | 0.74 | (0.53, 1.03) |  | 0.80 | (0.62, 1.03) | 0.495 |
| Sex (female) | 1.84 | (1.55, 2.18) |  | 1.26 | (1.08, 1.47) |  | 1.50 | (1.33, 1.70) | <0.001 |
| Maternal status (unmarried) | 1.51 | (1.24, 1.85) |  | 1.77 | (1.47, 2.12) |  | 1.64 | (1.42, 1.90) | 0.208 |
| Maternal education (low) | 1.20 | (0.92, 1.56) |  | 1.56 | (1.20, 2.03) |  | 1.38 | (1.13, 1.69) | 0.122 |
| Home ownership (rented) | 1.52 | (1.25, 1.86) |  | 1.66 | (1.37, 2.02) |  | 1.59 | (1.37, 1.85) | 0.504 |
| **Familial** |  |  |  |  |  |  |  |  |  |
| Family History of depression | 1.38 | (1.15, 1.66) |  | 1.11 | (0.94, 1.32) |  | 1.24 | (1.08, 1.42) | 0.057 |
| Family history of schizophrenia | 1.58 | (1.01, 2.47) |  | 1.32 | (0.82, 2.10) |  | 1.45 | (1.03, 2.04) | 0.561 |
| **Neurodevelopmental** |  |  |  |  |  |  |  |  |  |
| Asphyxia | 0.82 | (0.60, 1.12) |  | 1.26 | (0.96, 1.64) |  | 1.04 | (0.84, 1.29) | 0.028 |
| Gross Motor Levels | 1.03 | (0.98, 1.09) |  | 1.06 | (1.00, 1.12) |  | 1.05 | (1.01, 1.09) | 0.500 |
| Gross Motor Differentials | 1.00 | (0.93, 1.07) |  | 1.09 | (1.01, 1.17) |  | 1.04 | (0.99, 1.10) | 0.064 |
| IQ | 1.01 | (0.92, 1.10) |  | 0.88 | (0.81, 0.95) |  | 0.94 | (0.88, 1.00) | 0.018 |
| Autistic Spectrum Disorder | 0.96 | (0.76, 1.21) |  | 1.26 | (1.04, 1.54) |  | 1.11 | (0.95, 1.31) | 0.059 |
| **Stress and adversity** |  |  |  |  |  |  |  |  |  |
| Life Events | 1.14 | (0.98, 1.32) |  | 1.15 | (0.99, 1.33) |  | 1.15 | (1.02, 1.28) | 0.900 |
| Victimization | 1.35 | (1.12, 1.63) |  | 1.44 | (1.20, 1.74) |  | 1.40 | (1.21, 1.62) | 0.579 |
| **Emotional & behavioural** |  |  |  |  |  |  |  |  |  |
| Conduct | 1.17 | (1.07, 1.27) |  | 1.24 | (1.14, 1.36) |  | 1.21 | (1.13, 1.29) | 0.322 |
| Hyperactivity | 1.11 | (1.01, 1.21) |  | 1.22 | (1.12, 1.34) |  | 1.17 | (1.09, 1.25) | 0.097 |
| Peer problems | 1.17 | (1.07, 1.28) |  | 1.24 | (1.14, 1.36) |  | 1.21 | (1.13, 1.29) | 0.326 |
| Anxiety | 1.37 | (0.76, 2.49) |  | 2.01 | (1.21, 3.36) |  | 1.70 | (1.13, 2.57) | 0.297 |
| Depression ( at 12 years) | 3.01 | (2.36, 3.85) |  | 2.50 | (1.95, 3.21) |  | 2.75 | (2.26, 3.34) | 0.228 |
| PEs (at 12 years) | 2.24 | (1.77, 2.83) |  | 3.92 | (3.11, 4.96) |  | 3.02 | (2.53, 3.60) | <0.000 |
| **Substance use** |  |  |  |  |  |  |  |  |  |
| Cannabis use | 1.22 | (1.11, 1.33) |  | 1.20 | (1.08, 1.33) |  | 1.21 | (1.12, 1.30) | 0.790 |

^a^ p-values associated with significance tests comparing a model assuming psychopathology-specific effect for each exposure vs. a model where the exposure effect is common/shared (i.e. constrained to be the same across psychopathologies). Small p-values indicate evidence of differences in fit between the two models, whereby the shared-effect model does not provide adequate fit for the data and a psychopathology-specific one provides a better fit

**Supplementary Table S2:** Pooled estimates from multiple imputation (N = 7030) of exposure effects (OR and 95% CI) on depression and psychotic experiences (PEs) at 12 years and 18 years^a^, and examination of whether psychopathology-specific effects differ from a common effect across psychopathologies

|  | **Depression** | | | **PEs** | | | **Common effect** | |
| --- | --- | --- | --- | --- | --- | --- | --- | --- |
|  | **OR** | **95% CI** | | **OR** | **95% CI** | | **t-value** | **p-value^b^** |
| **Sociodemographic** |  |  |  |  |  |  |  |  |
| Ethnicity (non-White) | 1.28 | (0.98 | 1.68) | 1.20 | (0.92 | 1.57) | 0.390 | 0.694 |
| Urbanicty (rural) | 0.85 | (0.67 | 1.08) | 0.61 | (0.47 | 0.78) | 2.110 | 0.035 |
| Sex (female) | 1.69 | (1.50 | 1.89) | 1.18 | (1.06 | 1.32) | 5.040 | <0.001 |
| Maternal status (unmarried) | 1.37 | (1.20 | 1.57) | 1.63 | (1.42 | 1.86) | -1.990 | 0.047 |
| Maternal education (low) | 1.05 | (0.87 | 1.26) | 1.30 | (1.09 | 1.55) | -1.930 | 0.054 |
| Home ownership (rented) | 1.30 | (1.13 | 1.51) | 1.50 | (1.30 | 1.72) | -1.510 | 0.132 |
|  |  |  |  |  |  |  |  |  |
| **Familial** |  |  |  |  |  |  |  |  |
| Family History of depression | 1.27 | (1.13 | 1.44) | 1.22 | (1.09 | 1.37) | 0.560 | 0.575 |
| Family history of schizophrenia | 1.43 | (1.03 | 2.00) | 1.10 | (0.78 | 1.56) | 1.220 | 0.222 |
| **Neurodevelopmental** |  |  |  |  |  |  |  |  |
| Asphyxia | 0.98 | (0.80 | 1.20) | 1.30 | (1.08 | 1.58) | -2.350 | 0.019 |
| Gross Motor Levels | 1.01 | (0.98 | 1.05) | 1.06 | (1.02 | 1.10) | -1.820 | 0.069 |
| Gross Motor Differentials | 1.02 | (0.97 | 1.07) | 1.07 | (1.01 | 1.12) | -1.370 | 0.169 |
| IQ | 0.95 | (0.90 | 1.01) | 0.88 | (0.83 | 0.94) | 2.030 | 0.043 |
| Autistic Spectrum Disorder | 1.09 | (0.93 | 1.27) | 1.25 | (1.08 | 1.44) | -1.500 | 0.133 |
| **Stress and adversity** |  |  |  |  |  |  |  |  |
| Life Events | 1.17 | (1.07 | 1.27) | 1.17 | (1.08 | 1.27) | -0.020 | 0.985 |
| Victimization | 1.42 | (1.25 | 1.61) | 1.43 | (1.26 | 1.61) | -0.060 | 0.954 |
| **Emotional & behavioural** |  |  |  |  |  |  |  |  |
| Conduct | 1.20 | (1.12 | 1.28) | 1.20 | (1.13 | 1.28) | -0.010 | 0.988 |
| Hyperactivity | 1.15 | (1.08 | 1.23) | 1.23 | (1.16 | 1.30) | -1.640 | 0.102 |
| Peer problems | 1.23 | (1.16 | 1.31) | 1.25 | (1.17 | 1.33) | -0.270 | 0.786 |
| Anxiety | 1.59 | (1.08 | 2.35) | 1.61 | (1.10 | 2.36) | -0.040 | 0.966 |

^a^ after pooling over time, and assuming no time-varying exposure effects; ^b^ p-values associated with significance tests comparing a model assuming psychopathology-specific effect for each exposure vs. a model where the exposure effect is common/shared (i.e. constrained to be the same across psychopathologies). Small p-values indicate evidence of differences in fit between the two models, whereby the shared-effect model does not provide adequate fit for the data and a psychopathology-specific one provides a better fit
